# Changes of triage by GPs during the course of prehospital emergency situations in a Norwegian rural community

**DOI:** 10.1186/1757-7241-21-89

**Published:** 2013-12-19

**Authors:** Sverre Rørtveit, Eivind Meland, Steinar Hunskaar

**Affiliations:** 1 Austevoll Municipality Health Services, Bekkjarvik, 5399, Norway; 2 Department of Global Public Health and Primary Care, University of Bergen, Post box 7804, Bergen, 5020, Norway; 3 National Centre for Emergency Primary Health Care, Uni Health, Uni Research, Kalfarveien 31, Bergen, 5018, Norway

**Keywords:** Triage, Emergency medicine, Prehospital, General practice, Epidemiology

## Abstract

**Background:**

Priority grade assessment according to urgency level of the patients (triage) is considered vital in emergency medicine casualties. Little is known of the experiences of pre-hospital emergency medicine triage performed by General Practitioners (GPs) in the community. In this study we bring such experiences from a Norwegian island community, with special emphasis on over- and undertriage.

**Methods:**

In the island municipality of Austevoll, Western Norway, where the GPs and the ambulance services both take part in all medical emergency cases, all these cases were recorded during a 2-year period (2005–2007). We compared the triage of the patients at the stage of the telephone reception of the incident, and the subsequent revision of the triage at the first personal examination of the patient.

**Results:**

236 emergency medical events were recorded, comprising 240 patients. Of these, 42% were downgraded between the stages (i.e. initially overtriaged), 11% were upgraded (i.e. initially undertriaged) and 47% remained in unchanged priority group. Of the diagnostic groups, acute abdominal cases had the highest probability of being upgraded between stages, while the aggregated diagnostic group of syncopes, seizures, intoxications and traumas had the highest probability of being downgraded. The principal reason for upgrading was lack of necessary information at the stage of call. In a minority of cases the upgrading was due to real patient deterioration between stages.

**Conclusions:**

In pre-hospital triage of emergency patients, downgrading happens between notification of events and actual patient examination in a substantial proportion. Upgradings of cases are considerably fewer, but the potential serious implications of upgrading warrants individual scrutiny of such cases.

## Introduction

Dispatch guidelines, urgency assessment and clinical triage have long been applied in the health services in order to prioritize among patients with acute serious illness or injury [1]. Such systems usually give priority to patients according to fulfillment of predefined anamnestic criteria and clinical signs. In most Western countries different formal guidelines, algorithms or triage systems have been implemented in emergency medical communication centers (EMCCs) receiving medical emergency calls, in on-scene pre-hospital settings, ambulance services, and in the hospital emergency rooms and wards. Such systems are in general believed to be effective and useful tools in emergency situations. However, the scientific basis for most systems seems to be weak [2-4]. Especially, we found no relevant studies concerning scientific documentation of results of primary care on-scene triage.

Pre-hospital triage of emergency patients is necessarily an inexact process and some degree of *overtriage* must generally be accepted [5]. This means that patients may be approached by higher speed and more personnel than needed, then subsequently determined to have less severe illness or injury, thus being downgraded. The cost is overuse of medical resources and skewed allocation of resources to other patients. On the other hand, *undertriage* represents potential risks for clinical outcome, as emergency situations may develop into life-threatening situations during the pre-hospital course.

In day to day practice in many pre-hospital situations, however, assessment and triage of patients are performed without use of a formal triage system. Instead, different kinds of health care personnel apply the best appraisal that one is capable of, based on personal competence and experience, and thus assign priority and risk in an informal manner. This is typically the situation in primary care, where general practitioners (GPs) or ambulance personnel on call outs attend an emergency scene, using spontaneous and intuitive synthesizing of the available information and background knowledge. This process may be called *intuitive* triage. Also, in the majority of primary care events, triage is not used to prioritize between patients, but for assessing the level of urgency for a single patient, so that mode and speed of adequate transport can be determined. Accordingly there is a lack of data about reliability and validity of such pre-hospital assessments of adequate level of response. It must be anticipated that intuitive triage will have uncertain accuracy and generate both under-triage and over-triage of patients, with subsequent changes of priority grade along the course.

In a recent study we analyzed all patients managed by the pre-hospital emergency services in a Norwegian community during a two-year period [6,7]. Here, the triage was done by the GPs in the community, who took part in all the emergency situations both during office hours and during on-call out-of-hours. In the present study we have performed a post hoc analysis of our data in order to investigate changes in triage level during the course of the situation, based on assessments at two stages. Using the response categories of The Norwegian Index for Medical Emergency Assistance (Index) [8] the doctors’ initial assessment at telephone reception of the incident was recorded, and then again after clinical examination when seeing the patient. We could thus compare emergency patients with unchanged priority grade with those who developed a more or less serious condition (upgraded or downgraded patients). We examined the changes of priority grade according to diagnostic group, age and sex, and other factors. We especially explored the events which were upgraded to the highest level of response with regard to reason and possible learning implications for GPs in similar situations.

## Materials and methods

The study design and main results of the project evaluating emergency medical events in Austevoll municipality over a period of two years (2005–2007) have been published elsewhere [6,7]. Austevoll is a Norwegian island municipality south of Bergen without a mainland connection with 4 389 inhabitants per 1 January 2007. Ferries or express boats are used for ordinary transport to the mainland. Four GPs were on call during daytime and participated in out-of-hours services. Austevoll has one ambulance car and one ambulance boat and the personnel on the car and boat have 24-hour duties in a central ambulance station. In most cases, ambulance transport to hospital occurs by the ambulance car bringing the patient to the ambulance boat for further transport to a location on the mainland. Air ambulance (helicopter) with emergency care specialist contributed in about 10% of the events.

All activity associated with medical emergencies in Austevoll in the period October 2005 through September 2007 was recorded, with an exception of acute psychiatric events and patients giving birth. All emergencies were recorded, irrespective of whether the notification was made by the EMCC (which comprised for a little more than one third of the events) or more local instances. A medical emergency was defined as an event for which the doctor on call, based on the first notification, found the acuteness of the incident serious enough to see the patient without any delay. Information was also recorded for events which the doctor initially assessed as less serious, but for which clinical examination provided information that would have led to a call out, if the first notification had given the doctor this information.

For every medical emergency the doctor and ambulance personnel completed a registration form immediately after the event. The form was available electronically and on paper. Doctors were asked 84 questions and ambulance personnel 29 questions about clinical and practical aspects of the event. Upon notification of the event, the GP triaged the situation on the basis of all available information and background knowledge, irrespective of classifications made by the EMCC operator or others. The GP used the same categories as Index [8], but by using intuitive triage and not the formal system of Index, consisting of 40 different symptom cards with specific criteria that determine the response dispatched. Index divides the response into three categories; red, yellow and green. Red response is when the situation is acute and possibly life threatening, yellow response is for not life-threatening situations but requiring a doctor on site immediately, and green response is for non-urgent situations. The same triage grades were given by the same GP after examination of the patient on scene. For the purpose of this article we additionally collected data concerning some circumstances around the patient contact, like language problems, recording of whether there had been any direct contact between a GP and the patient for any serious disease during the last two months ahead of the emergency (comorbidity), or if there had been GP-patient-contact for the same diagnosis or a precursor of this diagnosis during the last two weeks of the emergency.

### Statistics

Differences between diagnostic groups were tested for statistical significance using cross tabulation and Pearson’s chi square tests. For analyses of cells containing less than five cases Fisher’s exact test was used. We subsequently adjusted for gender and age applying binary logistic regression analyses. Results were reported as ORs with 95% confidence intervals (CI). Age was recoded into three groups (0–20, 21–60 and >60 years). P <0.05 were considered statistically significant. SPSS version 18.0 was used.

### Ethics

The project was defined as a quality assurance project by the Norwegian Social Science Data Services, and thus not reported to the Regional Committee for Medical and Health Research Ethics. It was reported to the Norwegian Data Inspectorate, according to the Norwegian Health Registry Act.

## Results

A total of 236 emergency medical events were recorded, involving 240 patients (mean and median age 51 years (SD 28), 107 (45%) female). The majority of the events (84%) were caused by acute illness and the rest by injuries. GPs’ assessment of priority grade upon notification of emergencies yielded 79 red responses, 142 yellow, and 17 green, while two patients were not classified.

101 of the 240 patients (42%) underwent a severity downgrading when seen by the doctor (initial overtriage), 26 (11%) were upgraded (initial undertriage), while the priority grade was not changed in 111 (47%) (Table 1). Of the 26 upgradings, 13 were upgradings from green response to yellow, four from green to red, and nine from yellow to red. Of the 101 downgradings, 44 were from yellow response to green, 37 from red to yellow, and 20 from red to green.

**Table 1:** Priority grade changes by aggregated diagnostic groups

			**Priority grade changes**						**P-values**	
**Diagnostic group**	**Total**		**Unchanged**		**Upgraded**		**Downgraded**		**Upgraded**	**Downgraded**
	**%**	**N**	**%**	**N**	**%**	**N**	**%**	**N**		
Cardiac and cerebrovascular diseases*	100	86	56	49	10	9	32	28	0.864	0.020
Syncope, seizures, trauma, intoxication	100	76	39	30	3	2	57	44	0.004	0.001
Respiratory distress	100	30	50	15	13	4	37	11	0.753	0.494
Acute abdomen#	100	19	42	8	37	7	21	4	<0.001	0.036
Others	100	27	33	9	15	4	52	14	0.511	0.293
Total	100	238	47	111	11	26	42	101		

Pearson’s chi-square tests performed for upgrading and downgrading of each diagnostic group compared with all patients not belonging to that group. Fisher’s exact test if number in cell was < 5. Not relevant cases were excluded from statistical analyses (N = 2).

*Exclusive syncope and seizures.

#Inclusive gastrointestinal haemorrhage.

The priority grade changes by aggregated diagnostic groups are also shown in Table 1. In crude analyses, patients with acute abdominal symptoms had a significantly higher probability than other patients of being upgraded in priority when seen by the GP, while there was a statistically significant chance for the acute abdomen patients of not being downgraded. Patients with syncope, seizures, intoxication and trauma (aggregated group) had a significant higher probability than other patients of being downgraded, and a significantly reduced probability of being upgraded. Patients with cardiac and cerebrovascular diseases had a significant but small probability of not being downgraded in the second stage.

With logistic regression we tested the impact of gender and age groups on the probability of upgrading and downgrading (Table 2). There was a significant trend of upgrading by increasing age (p = 0.029), other age comparisons were not statistically significant. Men had a bordeline significant increased probability of being upgraded, compared to women (p = 0.050). The regression analyses confirmed the findings from the stratified analyses, except for acute abdominal symptoms, this group was now statistically insignificant for the probability of not being downgraded.

**Table 2:** Priority grade changes according to aggregated diagnostic groups

	**Upgrading**			**Downgrading**		
**Diagnostic group**	**Odds ratio**	**95% CI**	**P-value**	**Odds ratio**	**95% CI**	**P-value**
Cardiac and cerebrovascular diseases	0.775	0.311–1.932	0.585	0.535	0.294–0.975	0.041
Syncope, seizures, trauma, intoxications	0.131	0.030–0.582	0.007	2.667	1.469–4.843	0.001
Respiratory distress	1.179	0.365–3.809	0.784	0.549	0.228–1.324	0.182
Acute abdomen	5.525	1.880–16.23	0.002	0.325	0.104–1.010	0.052
Others	1.755	0.529–5.824	0.358	1.623	0.713–3.693	0.249

Logistic regression analyses, with adjustments for gender and age.

Table 3 shows the principal reasons for the upgradings (n = 26). For six of the 13 patients upgraded to red, clinical findings during the GP’s examination prompted the upgrading. In retrospect, we concluded that a higher response level was warranted already at the time of the emergency call in seven patients (five of them were upgradings to red), if it had been possible to adequately summarize all information at that stage. Real patient deterioration between the two stages seemed to have happened in six patients among all upgradings (23%), of whom two were upgradings to red.

**Table 3:** Principal reasons for upgrading of priority grade between the initial call and examination of the patient

	**Upgradings to red**		**All upgradings**		
**Reason**	**N**	**%**	**N**	**%**	
Patient examination necessary for clarification	6	46	13	50	
Accurate information not collected or available	5	38	7	27	
Impairment between stages	2	15	6	23	
Total	13	100	26	100	

Of the 26 upgraded patients, nine had one or more consultations or home visits by a GP for serious disease during the two months ahead of the emergency. Six patients had been seen by the doctor for the same diagnosis, or a precursor of this diagnosis, during the last two weeks before the emergency. Two of the patients belonged to both categories. Altogether, 13 patients (50%) had been seen by a doctor for serious comorbidity and/or the same diagnosis or a precursor of it, in the near time span before the emergency incident. Language problems concerning the communication with the caller were not found to be a problem in any case.

An upgrading to the highest priority grade means that the GP unexpectedly found the patient in a state of possible danger for life when arriving at scene or after initial clinical assessment. Table 4 gives more detailed information of these 13 cases. Among them, cardiac diseases were most frequent, followed by acute abdomen, respiratory distress and trauma. Of the five patients with cardiac diseases, two had acute myocardial infarction, one had a serious arrhythmia and one unexpectedly developed cardiac arrest. Two of the cases with acute abdomen were patients with serious gastrointestinal tract haemorrhage, and the two trauma cases were patients with drowning and near-drowning from a fishing boat accident. Five of the 13 upgradings to red happened in patients younger than 50 years of age, while four were over 80 years. A series of learning experiences may be extracted from qualitative analyses of such cases.

**Table 4:** Qualitative data on the 13 cases upgraded to red priority grade

**Case**	**Age**	**Sex**	**Initial priority**	**Clinical status when examined**	**Clinical diagnosis**	**Reason for upgrading**	**Possible learning implication**
1	90	M	Green	Dyspnea, pulmonary congestion	Pulmonary edema	Clinical examination needed	Beware of acute dyspnea in elderly patients
2	83	M	Yellow	Chest pain, ECG shows ST-elevation	Myocardial infarction	Clinical examination needed	Beware of long-lasting “angina”
3	67	F	Yellow	Cardiac arrest	Cardiac arrest	Impairment between assessments	Acute cardiac symptoms may develop into cardiac arrest
4	72	M	Yellow	Pallor, tachycardia, sweating, ECG: ventricular tachycardia	Ventricular tachycardia	Clinical examination needed	Acute tachycardia is not necessarily of pre-ventricular origin
5	33	M	Yellow	Abdominal pain, abdominal wall tenderness	Acute abdominal pain	Clinical examination needed	Beware of confusion by information of familiar gastroenteritis
6	73	F	Yellow	Pre-shock because of gastrointestinal hemorrhage	Gastrointestinal hemorrhage	Information not collected or initially unavailable	Beware of signs of GI hemorrhage by call receipt
7	90	M	Yellow	Pre-shock because of gastrointestinal hemorrhage	Gastrointestinal hemorrhage	Information not collected or initially unavailable	Beware of signs of GI hemorrhage by call receipt
8	31	M	Yellow	Chest pain, dyspnea, tachypnea	Pulmonary embolism?	Clinical examination needed	Beware of combination of acute dyspnea and chest pain
9	41	F	Green	Drooling, unable to swallow, near-obstruction of fauces	Acute epiglottitis?	Clinical examination needed	Beware of excessive swallowing problems and hyperpyrexia
10	45	M	Green	Somnolence/stupor	Somnolence	Information not collected or initially unavailable	Beware of information of multiple consciousness absences
11	89	M	Green	Respiratory insufficiency. Parkinson’s disease and pneumonia	Pneumonia and Mb Parkinson	Impairment between assessments	Parkinson’s disease and pneumonia: Increased risk of respiratory failure
12	62	M	Yellow	Patient was drowned because of boat accident, resuscitation failed	Drowning	Information not collected or initially unavailable	Correct information gathering is essential in trauma
13	37	M	Yellow	Same boat accident, had swam to land. Hypothermia	Drowning	Information not collected or initially unavailable	Correct information gathering is essential in trauma

## Discussion

In this two-year observational study of 240 medical emergency patients in an island community of Norway we found that severity priority grade was downgraded from call reception to after patient examination in 42%, representing a large volume of overtriage. Upgradings were much less frequent (11%), although half of these cases were to red (acute) priority grade, thus representing undertriage and a potential for harm. In about one out of four the upgrading was due to deterioration of the clinical condition, and could not have been initially detected. Upgrading was frequent in acute abdomen, while downgrading was particular frequent in the aggregated diagnostic group of syncope, seizures, intoxication and trauma.

The strength of our study is that we present population based data from a comprehensive and consecutive collection of all medical emergencies in a Norwegian community. The study also enabled us to compare GPs’ triage of all the patients at two stages, so that we could evaluate magnitudes and characteristics of priority changes (overtriage and undertriage) in a real-world patient setting. We have not found similar studies from a primary care emergency medicine setting.

GPs involvement in community emergency service varies, and the involvement in Austevoll may be greater than in the average municipality in Norway. In spite of this, we maintain that our findings are valid and generalizable, because participating GPs differed in experience and competence.

Weaknesses of our study include that we do not have any information of what was the final diagnosis and seriousness of the patients who were admitted to hospital. Further, a formal triage system was not applied, as the triage was done as intuitive triage. However, all the GPs used the categories from Norwegian index for medical emergency assistance [8], which they all knew reasonable well, especially the main response categories red, yellow and green, which they use in daily practice when communicating with both local emergency medical communication centers and the hospital based EMCC. Another weakness of the study is the relatively few cases that were upgraded. This induces the potential of lack of statistical power in some analyses, and relevant differences may thus not have reached statistical significance (type II error). Lastly, GPs may have been biased by the knowledge of the examination triage when they assigned the notification triage. This bias probably led to an underestimation of the difference between primary and final evaluation.

Downgrading of syncope, seizures, intoxication and trauma cases may be explained by the often experienced dramatic messages that are conveyed in such cases in the initial communication process, while most such cases turn out to be less serious when encountered and examined. The same sequence of reduced perception of seriousness between stages makes upgrading significantly low-frequent for this combined diagnostic group. Cardiac and cerebrovascular disorders had a small, but statistically significant, reduced probability of being downgraded. This might be because the GPs at the moment of patient examination did not feel confident to rule out serious conditions like myocardial infarction or stroke.

The detected undertriage of acute abdomen cases was a bit unexpected, and may be a finding of importance for GPs on call in out-of-hours services. Especially, an initial call of an acute abdomen case together with a suspicious history or symptoms for gastrointestinal bleeding should warrant high attention.

We found frequent upgradings in cases where the patient recently had an encounter with a GP**.** When patients readmit with possible alarming symptoms after a recent evaluation, this might be an indication of serious acute disease.

Concerning the upgradings to red response, the examination of the qualitative data shows that in most cases we found no significant deterioration in the time course between initial call and examination. However, in most cases the real priority grade was not possible to assess until clinical assessment, examination and e.g. ECG was performed, and the GP could summarize the situation into a final conclusion. In some cases, however, it would have been possible for the GP to comprehend the real priority grade from the moment of call reception, if she or he had penetrated the available anamnestic data in a better way, or had had the possibility of obtaining better information during initial assessment by telephone. Based on our findings there is thus a potential for reducing both undertriage and overtriage, although we are aware that a reasonable portion of over-triage must be accepted as we have shown that a doctor’s competence and skills at scene often are necessary to analyze the clinical situation accurately [7,9].

We were especially interested in any learning messages from cases where the patient unexpectedly was found in a possible life-threatening situation. Indications of gastrointestinal bleeding deserve closer attention from the first doctor. The combination of dyspnoea and other risk factors also warrants high priority. The same is true when information is unclear as to what has happened with injured patients. A post-hoc audit and discussion of such cases among doctors and ambulance personnel may be useful.

In general, there are few studies that evaluate the effectiveness and reliability of triage systems for pre-hospital emergency services. The Index [8] used as a basis for our study has not been validated. In 2011 Dutch researchers published a systematic review of the scientific documentation of the safety of telephone triage in Western out-of-hours call centers [2] different triage systems were not investigated or compared in the study, and in the review it is not specified whether a triage system was applied in each of the studies making up the review. The conclusion of the review was that there is room for improvement in safety of telephone triage in patients who present symptoms that imply high risk. In 2011 also the Norwegian Knowledge Center for the Health Services performed a comprehensive search for studies, reviews and meta-analyses in order to evaluate the effectiveness and reliability of triage systems for the pre-hospital emergency services [3]. It was not possible to find studies containing data of sufficient quality to make a meaningful evaluation. In 2010 the Swedish Council on Health Technology Assessment evaluated the seven most applied triage systems then in use in Swedish hospital emergency departments [4]. They were not able to confirm the internal validity or the evidence that triage systems are effective in terms of medical outcomes. The single exception was that assigning the lowest degree of risk to the patients effectively predicted a benign patient outcome.

It is thus not possible, based on scientific evidence, to claim that any formal triage system would perform better than available clinical appraisal in a primary care emergency scene (*intuitive* triage by GPs). We have shown that both undertriage and overtriage occur in such situations, but it is neither possible to assess or specify the magnitude or implications of these inaccuracies nor can we compare the informal and formal systems.

In conclusion, in this comprehensive analysis of all medical emergency cases of a Norwegian rural community during two years, we found that acute abdomen cases had a significantly higher probability of being upgraded in priority, while the combined diagnose group of syncope, seizures, intoxication and trauma had a higher probability of being downgraded. Upgrading of cases are considerably fewer than downgradings, but the potential serious implications of upgrading warrants individual scrutiny of such cases.

## Competing interests

None of the authors have anything to declare.

## Authors’ contributions

SR has conceived the study, written the draft and revisions of the manuscript, performed the statistical analyses and approved the final version of the manuscript. EM and SH have contributed substantially to its content and several revisions. All authors take the responsibility for the paper as whole. All authors read and approved the final manuscript.
